# Phosphonylated tyrosine and cysteine disulfide adducts both generated from immunoglobulin G and human serum albumin indicate exposure to the nerve agent VX in vitro

**DOI:** 10.1007/s00216-025-05762-x

**Published:** 2025-02-01

**Authors:** Henrik Reuter, Dirk Steinritz, Franz Worek, Harald John

**Affiliations:** Bundeswehr Institute of Pharmacology and Toxicology, Neuherbergstr. 11, 80937 Munich, Germany

**Keywords:** Mass spectrometry, Organophosphorus nerve agent, Protein adducts, Solid-phase extraction

## Abstract

Pronase-catalyzed proteolysis is shown to produce single amino acid adducts of tyrosine (Tyr) and cysteine (Cys) obtained from both human serum albumin (HSA) and immunoglobulin G (IgG) after in vitro exposure of plasma to the nerve agent VX. Total plasma as well as isolated HSA and IgG yielded the Tyr residue phosphonylated with the ethyl methylphosphonic acid moiety, Tyr(*-EMP*). Furthermore, a Cys residue adducted with the diisopropylaminoethane thiol leaving group of the agent bound via a disulfide bridge, Cys(*-DPAET*), was also obtained from both proteins. Even though Tyr(-*EMP*) represents an internationally well-accepted biomarker of a VX-like agent its origin from plasma IgG has never been shown before. In addition, this is the first time that Cys(*-DPAET*) is presented as a biomarker of VX exposure clearly identifying the chemical nature of the V-type nerve agent’s leaving group. Both biomarkers were detected after selective affinity-based solid-phase extraction (SPE) from plasma that yielded highly purified HSA and IgG as documented by sodium dodecyl polyamide gel electrophoresis (SDS-PAGE). Both biomarkers were found in the corresponding protein bands of HSA and IgG each after in-gel proteolysis with pronase. A micro liquid chromatography-electrospray ionization high-resolution tandem-mass spectrometry method (LC-ESI HR-MS/MS) was developed for the simultaneous detection of Tyr(*-EMP*) and Cys(-*DPAET*). The time for proteolysis was optimized for maximum biomarker yield. The method showed excellent selectivity and sensitivity, and the adducted proteins and biomarkers were found to be highly stable during storage. Accordingly, the presented method sheds more light on the molecular toxicology of VX and broadens the spectrum of methods suited for biomedical verification.

**Graphical Abstract:**

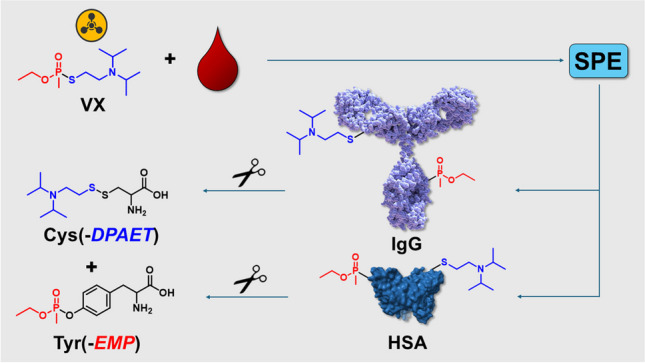

## Introduction

Organophosphorus nerve agents (OPNA) exhibit extremely high acute toxicity posing a serious threat to military and civilian personnel in strategic or terroristic attacks. Their toxicity is caused by the inhibition of the enzyme acetylcholinesterase (AChE) resulting from the phosphylation (denominating both phosphorylation and phosphonylation) of its active site serine residue [1, 2]. AChE inhibition induces the uncontrolled accumulation of acetylcholine in the synaptic cleft, provoking a cholinergic crisis that is characterized by miosis, hypersalivation, vomiting, and seizures, and might lead to death by respiratory failure [1–3]. The use of OPNA was observed in the recent past in assassinations and attempted murders in Japan [4–6] and the UK [7, 8], of a Russian dissident [9], and in warfare in Syria [10]. Nevertheless, according to the Chemical Weapons Convention (CWC), the deployment, production, and stockpiling of chemical warfare agents (CWA) are strictly prohibited [11]. Compliance with these regulations is supervised by the Organisation for the Prohibition of Chemical Weapons (OPCW, The Hague/The Netherlands, Nobel Peace Prize laureate 2013), supported by an international network of specialized laboratories, qualified, and designated for the analysis of environmental and biomedical samples.

**Fig. 1:**
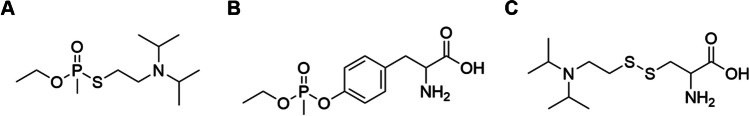
Chemical structures of VX and the adducted single amino acids. **A** Nerve agent VX (*S*−2-diisopropylaminoethyl *o*-ethyl methylphosphonothioate); **B** tyrosine phosphonylated by the ethyl methylphosphonyl-moiety, Tyr(-*EMP*) ([M + H]^+^ *m/z* 288.0995); **C** disulfide adduct of cysteine with the 2-diisopropylaminoethanethiol (*DPAET*) leaving group of VX, Cys(-*DPAET*) ([M + H].^+^ *m/*z 281.1352)

VX (Fig. 1A) is an OPNA that belongs to the group of V-type nerve agents that was used for the assassination of Kim Jong-nam in Malaysia in 2017 [6], highlighting the need for comprehensive analytical methods for the detection of VX. Its covalent reaction products with endogenous proteins (protein adducts) are typically analyzed to verify exposure to the agent [12]. Adducts result from the phosphonylation of nucleophilic moieties in the side chains of amino acids like the hydroxy group in the active site serine residue in AChE [12–14] as well as in butyrylcholinesterase (BChE) [12, 15]. Furthermore, tyrosine (Tyr) residues in, e.g., human serum albumin (HSA) [2, 12] and lysine (Lys) residues in, e.g., hair keratins and ubiquitin were also shown to be phosphonylated [16, 17]. In addition, disulfide adducts between the thiol-containing leaving group of VX (2-diisopropylaminoethanethiol, *DPAET*) and the cysteine residue at position 34 (Cys^34^) of HSA are used to document exposure to VX [12, 18–20]. These protein adducts are subjected to proteolysis with appropriate enzymes to produce adducted peptides or single amino acids, representing well-traceable biomarkers [12, 18–20]. Accordingly, exposure to VX is proven by the presence of Tyr adducts containing the ethyl methylphosphonic acid moiety (*EMP*), Tyr(-*EMP*) (Fig. 1B), and the dipeptide Cys^34^(-*DPAET*)Pro [12, 18–20]. Analyses might be carried out by microbore liquid chromatography-electrospray ionization high-resolution tandem-mass spectrometry (μLC-ESI HR-MS/MS) operating in the product ion scan mode (PIS) [12]. Tyr(-*EMP*) obtained from pronase-catalyzed cleavage of plasma proteins is most often attributed to originate from HSA [12], as it represents the most abundant protein in plasma (40 mg/mL). In contrast, immunoglobulins of the G class (IgG), representing the second most abundant group of proteins (7–18 mg/mL) [21], were not shown as an origin of single amino acid adducts so far.

IgG is part of the humoral immune system that mediates response to bacteria, viruses, and cellular antigens [22]. IgG is a 150-kDa glycoprotein comprised of a highly conserved constant region (F_c_, signaling cellular immune response) and two highly variable antigen-binding regions (F_ab_, selectively interacting with antigens) [23].

We postulated that pronase used for protein adduct cleavage will not only degrade HSA but also IgG thus forming single amino acid adducts. In this study, we therefore investigated human plasma exposed to VX in vitro and originally targeted IgG besides HSA to monitor the formation of Tyr(-*EMP*) (Fig. 1B) as well as of Cys(-*DPAET*) (Fig. 1C).

## Materials and methods

### Chemicals and reagents

Individual human EDTA plasma was from in.vent Diagnostica (Hennigsdorf, Germany) and pooled EDTA plasma from DUNN Labortechnik GmbH (Asbach, Germany). Pronase from *Streptomyces griseus* was from Roche (Mannheim, Germany), globulin-free HSA (≥ 99%), phosphate-buffered saline (PBS) tablets, and cysteine (97%) were from Sigma-Aldrich (Steinheim, Germany). Purified human IgG from serum (≥ 80%), acetonitrile (ACN, LC grade), water (LC grade), ultrafiltration (UF) devices (Amicon Ultra-0.5 centrifugal filter unit, 0.5 mL, molecular weight cutoff, MWCO, 10 kDa), and ProteoExtract® albumin/IgG removal kits, maxi for affinity-based solid-phase extraction (SPE) were from Merck (Darmstadt, Germany), triple deuterated atropine (*d*_3_-Atr) was from CDN Isotopes (Pointe-Claire, Quebec, Canada), NH_4_HCO_3_ from Fluka (Buchs, Switzerland), and formic acid (FA) from Carl Roth (Karlsruhe, Germany). The bicinchoninic acid (BCA) assay was carried out using the BCA assay protein quantitation kit from Advion Interchim Scientific (Montluçon, France). NuPAGE™ 4–12% Bis–Tris gels used for non-reducing sodium dodecyl sulfate–polyacrylamide gel electrophoresis (SDS-PAGE) were from Thermo Fischer Scientific (Waltham, MA, USA). The nerve agent VX (CAS no. 50782–69-9) was provided by the German Ministry of Defense and was tested for integrity and purity in-house by NMR spectroscopy. A stock solution of 37.4 mM (10 mg/mL) was prepared in acetonitrile (ACN), and working solutions were produced by serial dilution with ACN yielding VX concentrations of 10 mM, 5 mM, 2.5 mM, 1.25 mM, 625 µM, 312 µM, 156 µM, 78.1 µM, 39.1 µM, 19.5 µM, 9.77 µM, 4.88 µM, 2.44 µM, 1.22 µM, and 0.61 µM. All other chemicals were from common providers.

### Incubation of plasma and neat proteins with VX

Incubations of plasma and neat proteins were carried out as follows to produce references: Plasma, neat IgG solution (13.5 mg/mL in PBS), and neat HSA solution (40 mg/mL in PBS) (990 µL, each) were mixed with the stock solution of VX (10 µL), yielding final VX concentrations of 374 µM for 24-h incubation under gentle shaking at 37 °C. Incubations carried out with other VX concentrations mentioned below were carried out under the same conditions. Blanks were prepared accordingly using neat ACN instead of VX solutions.

### Synthesis of adducted cysteine as analytical standard

Synthesis of adducted cysteine was carried out similar to that of adducted tyrosine as described before [16]. In brief, cysteine (10 μL, 72.5 μg/mL in 50 mM NH_4_HCO_3_) was mixed with the stock solution of VX (50 μL) for a 24-h incubation at 37 °C under gentle shaking. Afterwards, an aliquot (2 μL) was diluted with *d*_3_-Atr solution (238 μL, 3 ng/mL in 0.5% *v*/*v* FA) prior to μLC-ESI HR-MS/MS (PIS) analysis to detect and fragment Cys(-*DPAET*) ([M + H]^+^ *m/z* 281.1352).

### Proteolysis of neat IgG, HSA, and total proteins in plasma

According to the standard protocol, proteolysis was carried out as follows: Proteins from incubation mixtures of IgG solutions, HSA solutions, and plasma (100 µL, each) were precipitated by the addition of ice-cold ACN (200 µL), followed by centrifugation (5 min, 18,620 RCF, 15 °C), removal of the supernatant, and washing the protein pellet two times with ice-cold ACN (200 µL). The pellet was suspended in NH_4_HCO_3_ (150 µL, 50 mM) and pronase solution (100 µL, 60 mg/mL in 50 mM NH_4_HCO_3_) and incubated at 42 °C for 2 h under vigorous shaking. Afterwards, the incubation mixture was transferred into an UF device (MWCO 10 kDa) for UF (10 min, 18,860 RCF, 15 °C). An aliquot of the filtrate (60 µL) was diluted with *d*_3_-Atr solution (120 µL, 3 ng/mL in 0.5% *v*/*v* FA) and analyzed by μLC-ESI HR-MS/MS (PIS) to detect the adducted biomarkers.

### Affinity-based SPE of plasma

Affinity-based SPE was carried out using the ProteoExtract® albumin/IgG removal kit, maxi according to the manufacturer’s instructions. In brief, plasma references and blank plasma (100 µL, each) were separately mixed with the supplied binding buffer (10 ×) (100 µL, 250 mM sodium phosphate, pH 7.4) and water (800 µL). The binding buffer (1 ×) was prepared by 1:10 dilution of the binding buffer (10 ×) with water. The column assembly consisting of an HSA-selective column connected with an IgG-selective column was equilibrated with binding buffer (1 ×) (4 mL) before diluted plasma (1 mL) was loaded onto the column and washed afterwards with binding buffer (1 ×) (4 mL). The flow-through fraction (1 mL) and the fraction of the washing step (4 mL) were combined and are denominated herein as depleted fraction. Afterwards, the column assembly was demounted and HSA was eluted from its column by using the supplied elution buffer blue (25 mM sodium phosphate, pH 8.0, 2 M NaCl, 4 mL). The first 2 mL was collected and is referred to herein as HSA fraction. IgG was eluted from its column with the supplied elution buffer protein A (250 mM citric acid, 4 mL). The first 2 mL was collected and is referred to herein as IgG fraction.

### Proteolysis of HSA and IgG from SPE fractions

IgG fractions and HSA fractions from affinity-based SPE (2 mL, each) were separately concentrated and subjected to buffer exchange by repeated UF (MWCO 10 kDa, 10 min, 18,860 RCF, 15 °C). Buffer exchange was achieved by adding NH_4_HCO_4_ (400 µL, 50 mM) to the retentate of each fraction (100 µL) in the UF device followed by UF (10 min, 18,860 RCF, 15 °C). This step was repeated three times. The final retentate (100 µL) was transferred into a reaction tube and the UF device was washed with NH_4_HCO_3_ (150 µL, 50 mM) to combine this rinsing solution with the final retentate (250 µL in total). The protein concentrations of these diluted retentates were determined by a BCA assay according to the manufacturer’s protocol to calculate protein recoveries. Pronase solution (100 µL, 60 mg/mL in 50 mM NH_4_HCO_3_) was added to the diluted retentates (240 µL) for proteolysis, followed by incubation at 42 °C for 2 h under vigorous shaking.

### Comparative measurement of adduct yield during proteolysis of SPE fractions

Affinity-based SPE of plasma references was carried out in nonuplicate (*n* = 9) to yield sufficient protein amounts for kinetic investigations. The retentates (240 µL, *n* = 9) of the IgG and HSA fractions were pooled separately and NH_4_HCO_3_ (300 µL, 50 mM) was added yielding a total volume of 2460 µL, each. The protein concentration of the pooled retentates was determined by a BCA assay and split into three aliquots (750 µL, each). For subsequent proteolysis (*n* = 3), pronase solution (300 µL, 60 mg/mL in 50 mM NH_4_HCO_3_) was added each followed by incubation at 42 °C under vigorous shaking. During proteolysis, aliquots (50 µL) were taken after 5 min, 10 min, 20 min, 30 min, 1 h, 1.5 h, 2 h, 3 h, 4 h, and 5 h, mixed with *d*_3_-Atr solution (100 µL, 3 ng/mL in 0.5% *v*/*v* FA) in UF devices and subjected to UF (10 min, 18,860 RCF, 15 °C). Analysis of the filtrates was done by μLC-ESI HR-MS/MS (PIS) to detect Tyr(-*EMP*) and Cys(-*DPAET*). Resulting peak areas of the qualifying ion I (Qual I) of Tyr(-*EMP*) and Cys(-*DPAET*) were used as a measure of the absolute yield (y_*abs*_) of biomarkers. Ratios of peak areas to the corresponding total amount of proteins in the prepared fraction (mass in mg) are denominated herein as the “mass yield” (y_*mass*_). Furthermore, the ratio of the peak areas to the molarity of proteins in the fractions was calculated as a measure of the molar yield (y_*mol*_) of biomarkers. The diverse yields obtained from triplicate analysis were plotted as mean and standard deviation (*M* ± SD) against the time of proteolysis to characterize the protein-dependent yield of biomarkers.

### μLC-ESI HR-MS/MS (PIS) analysis

Chromatographic separation of amino acid adducts was carried out at 35 °C on an Acquity UPLC HSS T3 C18 column (50 × 1.0 mm I.D., 1.8 µm, 100 Å, Waters, Eschborn, Germany) protected by a security guard™ ultra cartridge UHPLC precolumn (C18-peptide, 2.1 mm I.D., Phenomenex, Aschaffenburg Germany). A microLC 200 system (Eksigent Technologies LLC, Dublin, CA, USA) was used for gradient elution (30 µL/min) with solvent A (H_2_O, 0.05% *v*/*v* FA) and solvent B (ACN/H_2_O 80:20, 0.05% *v*/*v* FA): *t* [min]/B [%]: 0/4, 11/39, 11.5/95, 13.5/95, 14/4, 15/4. Prepared samples were injected (20 µL) into a sample loop (Sunchrom, Friedrichsdorf, Germany) by an HTC-xt DLW autosampler (CTC Analytics, Zwingen, Switzerland) with a sample tray kept at 15 °C. Chromatography was coupled to a hybrid triple quadrupole time-of-flight (TOF) mass spectrometer (TT5600^+^, Sciex, Darmstadt, Germany) via an electrospray ionization (ESI) interface operating in positive ionization mode. Calibration was done by infusion of a calibration solution (APCI positive calibration solution, Sciex) via a calibrant delivery system (Sciex) directly connected with an atmospheric pressure chemical ionization (APCI) inlet after every fifth run. The mass spectrometer operated in the PIS mode for nitrogen-mediated collision-induced dissociation (CID) of protonated biomarker ions of Tyr(-*EMP*) ([M + H]^+^ *m/z* 288.1) and Cys(-*DPAET*) ([M + H]^+^ *m/z* 281.1) Product ions were monitored in a mass range from *m/z* 50 to *m/z* 400 in high-resolution mode. The following MS parameters were the same for all analytes: ion spray voltage floating (ISVF) + 5000 V, ion source temperature (TEM) 200 °C, ion source gas 1 (GS1) 40 psi (2.76 × 10^5^ Pa), ion source gas 2 (GS2) 50 psi (3.45 × 10^5^ Pa), curtain gas (CUR) 30 psi (2.07 × 10^5^ Pa), declustering potential (DP) 60 V, collision energy spread 0 V, ion release delay 67 ms, and an accumulation time of 50 ms. The collision energy (CE) was set to 25 V for Tyr(-*EMP*) and Cys(-*DPAET*) and to 42 V for the internal standard *d*_3_-Atr (*m/z* 293.2). For data analysis, extracted ion chromatograms (XIC) of the three most intense product ions (qualifier ions, Qual I–III) were generated with a mass accuracy of ± 0.005 Th, each. Tyr(-*EMP*) was monitored with *m/z* 214.0628 (Qual I), *m/z* 242.0941 (Qual II), and *m/z* 197.0362 (Qual III). Cys(-*DPAET*) was monitored with *m/z* 151.9834 (Qual I), *m/z* 160.1154 (Qual II), and *m/z* 114.1277 (Qual III) (Table 1). The mass accuracy was calculated based on the difference between the measured (accurate) mass and the theoretical (exact) mass. The entire µLC-ESI HR-MS/MS system was controlled by the Eksigent Control 4.3 and Analyst TF 1.7.1 software and MS data was processed by PeakView 2.2.0 and Sciex OS 1.7.0 (all Sciex). Data visualization was done with GraphPad Prism version 10.2.3 (GraphPad Software, Boston, MA, USA).

**Table 1:**
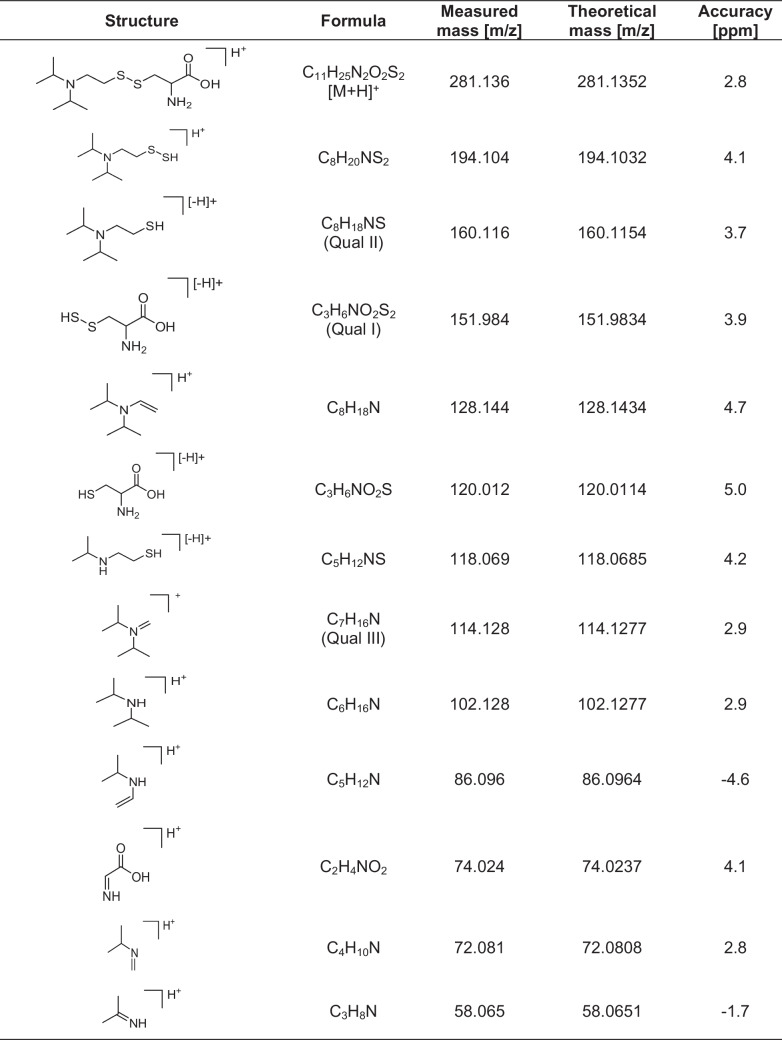
Precursor and product ions after collision-induced dissociation of single protonated Cys(*-DPAET*). [M + H]^+^: single protonated precursor ion, Qual I–III: qualifying ions I to III, for fragmentation, the mass of the precursor ion was set to *m*/*z* 281.1 and product ions were monitored in the range from *m/z* 50 to *m/z* 400

### Selectivity of biomarker detection

Blank plasma from six healthy individuals as well as solutions of neat IgG and neat HSA was prepared, proteolyzed, and analyzed by μLC-ESI HR-MS/MS (PIS) for the presence of interferences for Tyr(-*EMP*) and Cys(-*DPAET*) according to the standard protocol.

### Adduct yield during proteolysis of plasma

A plasma reference (500 µL) was precipitated and mixed with pronase solution according to the principles of the standard protocol (*n* = 3). During proteolysis, aliquots (50 µL) were taken after 5 min, 10 min, 20 min, 30 min, 1 h, 2 h, 3 h, 4 h, and 5 h. Aliquots were mixed with *d*_3_-Atr solution (100 µL, 3 ng/mL in 0.5% *v*/*v* FA) in UF devices and subjected to UF (10 min, 18,860 RCF, 15 °C). Analyses of the filtrates were done by μLC-ESI HR-MS/MS (PIS) to detect Tyr(-*EMP*) and Cys(-*DPAET*). Resulting peak areas of Qual I of Tyr(-*EMP*) and Cys(-*DPAET*) (*M* ± SD) were plotted against the time of proteolysis to determine the optimal incubation time for proteolysis.

### VX concentration-dependent adduct yield and limit of identification

Blank plasma (960 µL) was mixed with working solutions of VX (40 µL), yielding final VX concentrations of 400 µM, 200 µM, 100 µM, 50 µM, 25 µM, 12.5 µM, 6.25 µM, 3.12 µM, 1.56 µM, 781 nM, 391 nM, 195 nM, 97.7 nM, 48.8 nM, and 24.4 nM followed by 24-h incubation under gentle shaking. Precipitation, proteolysis, and µLC-ESI HR-MS/MS (PIS) analysis were carried out according to the standard protocol (*n* = 3, each). Resulting peak areas of Qual I to Qual III of Tyr(-*EMP*) and Cys(-*DPAET*) (*M* ± SD) were plotted against the VX concentration in plasma to characterize the linearity and the limit of identification (LOI). The LOI was defined as the lowest VX concentration still yielding traceable adducts fulfilling ion ratio criteria set by the OPCW for biomarker confirmation in forensic samples [24].

### Stability of protein adducts in plasma at 37 °C

Plasma references were stored at 37 °C under gentle shaking (*n* = 3) to analyze aliquots by μLC-ESI HR-MS/MS (PIS) according to the standard protocol after 15 h, 24 h, 40 h, and 63 h of storage. Peak areas of Qual I of Tyr(-*EMP*) and Cys(-*DPAET*) (*M* ± SD) were determined as measures of protein adduct stability.

### Stability of protein adducts after freeze-and-thaw cycles

Plasma references were prepared, proteolyzed, and analyzed by μLC-ESI HR-MS/MS (PIS) according to the standard protocol (*n* = 3) after incubation for 15 h at 37 °C and after three freeze-and-thaw cycles. Each cycle consisted of 24 h storage at − 30 °C followed by thawing, storage for 1 h at room temperature, sampling, and freezing again. Peak areas of Qual I of Tyr(-*EMP*) and Cys(-*DPAET*) (*M* ± SD) were determined as measures of protein adduct stability.

### Stability of biomarkers in the autosampler

Plasma references were prepared according to the standard protocol. The sample solution ready for injection was stored in the autosampler for 24 h at 15 °C and analyzed every hour by μLC-ESI HR-MS/MS (PIS). Resulting peak areas of Qual I were plotted against the time of storage to assess the stability of Tyr(-*EMP*) and Cys(-*DPAET*).

### SDS-PAGE analysis of SPE fractions

Aliquots of SPE fractions from plasma references and blank plasma were separated by non-reducing one-dimensional SDS-PAGE and stained with Coumassie Brilliant Blue. Bands corresponding to IgG and HSA were subjected to in-gel proteolysis with pronase following a common protocol as described earlier [16, 25] and analyzed for Tyr(-*EMP*) and Cys(-*DPAET*) by μLC-ESI HR-MS/MS (PIS).

### Safety considerations

VX must be handled by trained personnel only, wearing appropriate personal protection equipment. Work must be carried out in a fume hood and equipment which had contact to VX must be decontaminated. Decontamination was carried out by submerging the contaminated material in sodium hypochlorite (NaOCl) solution for 24 h at room temperature.

## Results and discussion

Established methods for the verification of VX exposure often make use of proteolysis of plasma proteins either directly in plasma matrix or after their precipitation without prior protein separation [12, 26–28]. Proteolysis with pronase yields the phosphonylated single Tyr residue that represents an internationally well-accepted biomarker of exposure [20, 29, 30]. In addition, disulfide adducts between Cys-containing peptides and the thiol-containing leaving group of the V-type nerve agent, e.g., Cys(*-DPAET*)Pro, MetProCys(*-DPAET*), ProCys(*-DPAET*), and AspIleCys(*-DPAET*), may also be used as biomarkers as already introduced by our working group [12, 18–20]. As monitoring of both biomarkers is not restricted to the identification of the phosphyl-moiety alone as known for, e.g., BChE adducts [12], the simultaneous detection allows the identification of the respective entire agent. Therefore, the simultaneous detection of Tyr and Cys adducts represents a major advantage for biomedical verification. So far, only HSA-derived Cys-peptides as mentioned above were introduced in the literature [12, 18–20]. Adducted single Cys residues have not been described before even though pronase often applied for proteolysis favors the degradation of proteins down to single amino acids. Therefore, we hypothesized that in addition to phosphonylated Tyr residues single Cys residues adducted with the leaving group should also be obtained after pronase-catalyzed proteolysis of VX-exposed plasma proteins. VX was tested exemplarily as it represents the most common and a very well characterized agent of the V-type nerve agent group. Accordingly, VX-exposed neat plasma proteins as well as total plasma were subjected to enzymatic cleavage and prepared samples were analyzed by targeted µLC-ESI HR-MS/MS (PIS) to prove the presence of Tyr(*-EMP*) and Cys(-*DPAET*). Synthetic standards were initially produced and analyzed to fix the chromatographic and mass spectrometric properties and to confirm biomarkers from the biological plasma matrix.

### μLC-ESI HR-MS/MS (PIS) detection of Tyr(*-EMP*) and Cys(*-DPAET*)

**Fig. 2:**
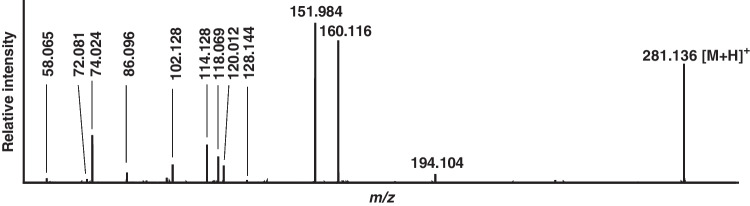
HR-MS/MS analysis of Cys(-*DPAET*) standard. The disulfide adduct was obtained by direct chemical reaction of VX with cysteine. All signals labelled were assigned to product ion structures as listed in Table 1

Tyr(*-EMP*) (Fig. 1B) is a well-characterized biomarker whose HR-MS/MS spectrum was already introduced in the literature [16]. In contrast, no data was available for Cys(*-DPAET*) (Fig. 1C), so far. Therefore, Fig. 2 now illustrates its corresponding HR-MS/MS spectrum obtained from the synthetic standard. As summarized in Table 1, the standard allowed the structural assignment of a large number of product ions with excellent accuracy (ppm < 5) even for ions with relatively low abundance.

**Fig. 3:**
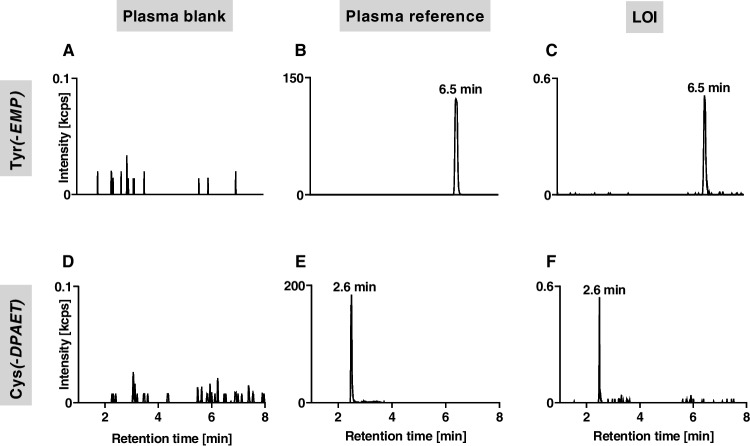
Chromatograms of Tyr(-*EMP*) and Cys(-*DPAET*) obtained after pronase-catalyzed cleavage of total plasma proteins. Analysis of Tyr(-*EMP*) in **A** plasma blank, **B** plasma reference, and **C** plasma spiked at LOI level, VX concentration 781 nM (209 ng/mL) and analysis of Cys(-*DPAET*) in **D** plasma blank, **E** plasma reference, and **F** plasma spiked at LOI level, VX concentration 195 nM (52.2 ng/mL). Extracted ion chromatograms were obtained from microbore liquid chromatography-electrospray ionization high-resolution tandem-mass spectrometry (µLC-ESI HR-MS/MS) analysis operating in product ion scan mode (PIS). For reasons of clarity, only traces of the most intense product ions of Tyr(-*EMP*) (*m/z* 214.063 ± 0.005 Th, Qual I) and of Cys(-*DPAET*) (*m/z* 151.983 ± 0.005 Th, Qual I) are shown

Considering these chromatographic and tandem-mass spectrometric characteristics of Tyr(*-EMP*) and Cys(*-DPAET*), we then succeeded in the detection of these adducts from VX-exposed neat plasma proteins as well as in plasma. After precipitation and pronase-catalyzed cleavage of plasma proteins, the µLC-ESI HR-MS/MS (PIS) analysis proved the presence of Tyr(-*EMP*) at a retention time (*t*_R_) of 6.5 min (Fig. 3B) as well as of Cys(-*DPAET*) at *t*_R_ 2.6 min (Fig. 3E). Based on these findings, the formation and detection of both biomarkers were characterized in vitro to elaborate an optimized protocol (standard protocol) for total plasma analysis. Accordingly, the selectivity of the assay, the time course of biomarker formation during proteolysis, the VX concentration-dependent biomarker yield, and the stability of protein adducts during storage and of the biomarkers in the autosampler were investigated.

### Selectivity of biomarker detection

Individual blank plasma of six donors as well as neat IgG and neat HSA did not show any interference for Tyr(-*EMP*) and Cys(-*DPAET*) as exemplarily illustrated in Fig. 3A and D, thus indicating high selectivity of the method.

### Adduct yield during proteolysis of plasma

**Fig. 4:**
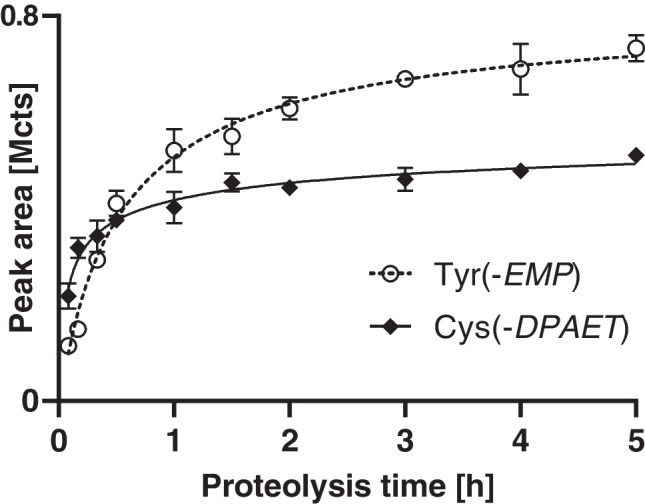
Time-dependent biomarker yield during proteolysis of total plasma exposed to VX. Biomarker yield was monitored during proteolysis recording Qual I of Tyr(-*EMP*) (*m/z* 214.063 ± 0.005 Th, open circles, dotted line) and Cys(-*DPAET*) (*m/z* 151.983 ± 0.005 Th, filled diamonds, solid line). Data points represent *M* ± SD of triplicate proteolysis.

Optimum biomarker signal intensity is of essential relevance for trace analysis, thus requiring an optimum incubation time for proteolysis to ensure maximum biomarker yield. After 2 h of proteolysis of proteins in total plasma, stable concentrations were found for both Tyr(-*EMP*) and Cys(-*DPAET*) (Fig. 4). Therefore, a 2-h incubation time was fixed for the standard protocol. Cys(-*DPAET*) was formed more rapidly than Tyr(-*EMP*), indicating that the majority of adducted Cys residues might be better accessible for proteolytic cleavage by pronase than Tyr adducts.

### VX concentration-dependent adduct yield

**Fig. 5:**
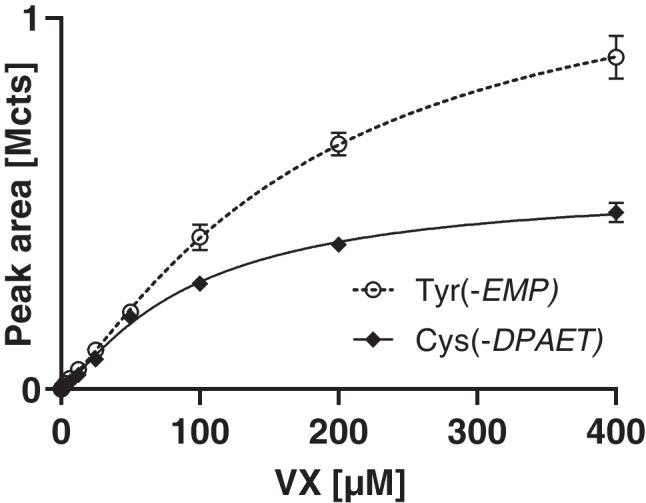
VX concentration-dependent adduct yield of Tyr(-*EMP*) and Cys(-*DPAET*) obtained from total plasma. Plasma was incubated with VX concentrations ranging from 24.4 nM to 400 µM (6.53 ng/mL to 107 µg/mL). The biomarker yield was monitored recording Qual I of Tyr(-*EMP*) (*m/z* 214.063 ± 0.005 Th, open circles, dotted line) and Cys(-*DPAET*) (*m/z* 151.983 ± 0.005 Th, filled diamonds, solid line). Data points represent *M* ± SD of triplicate incubation of plasma with VX

Over the entire range of VX concentrations tested from 24.4 nM to 400 µM in plasma (6.53 ng/mL to 107 µg/mL), both biomarkers were traceable (Fig. 5). The Tyr(-*EMP*) peak area rose over the entire concentration range (Fig. 5, open circles) starting with an initial linearity between 97.7 nM and 50 µM (26.1 ng/mL to 13.4 µg/mL; 97.6 nM to 50 µM; *R*^2 ^= 0.9969). The Cys(-*DPAET*) peak area also followed an initial linear increase (24.4 nM to 12.5 µM; 6.53 ng/mL to 3.34 µg/mL; *R*^2 ^= 0.9976) and was close to a plateau at VX concentrations above 200 µM (53.5 µg/mL) (Fig. 5, filled diamonds). The LOI was found at 195 nM VX (52.2 ng/mL) for Cys(-*DPAET*) (Fig. 3F, ion ratio Qual II/Qual I 88.4%, Qual III/Qual I 33.0%), thus proving Cys(-*DPAET*) as a highly sensitive marker of exposure. The LOI of Tyr(-*EMP*) was found at 781 nM (209 ng/mL) (Fig. 3C, ion ratio Qual II/Qual I 63.4%, Qual III/Qual I 30.0%). The lower LOI for Cys(-*DPAET*) correlated to earlier findings for disulfide adducts that cause a higher mass spectrometric response due to the favorable protonation of the basic adducted leaving group of VX (Fig. 1C) [18, 31].

### Stability of protein adducts in plasma at 37 °C

During storage of plasma references at 37 °C for 63 h, no degradation of biomarkers was observed, indicating high stability of protein adducts (results not shown).

### Stability of protein adducts during freeze-and-thaw cycles

During three freeze-and-thaw cycles, no decrease of the Tyr(-*EMP*) and Cys(-*DPAET*) yield was observed. Respective peak areas were found with a relative standard deviation (RSD) < 7%, each, thus also indicating appropriate stability of the protein adducts.

### Stability of biomarkers in the autosampler

During the storage period of 24 h at 15 °C in the autosampler, the peak areas of both biomarkers (Qual I) were stable (RSD < 7%) indicating high stability of the biomarker molecules.

The results presented above underline the potential of Cys(-*DPAET*) to be used as an additional biomarker besides Tyr(*-EMP*) to verify the exposure of plasma to VX in vitro. The value of this marker for human in vivo samples could not be elaborated so far due to the lack of samples from real case poisoning scenarios. Nevertheless, we made some efforts to further characterize these adducts with respect to their origin from plasma proteins.

Tyr(*-EMP*) is often discussed to be produced from HSA only, which is present in plasma as the most abundant protein. To figure out whether the IgG fraction, which represents the second most abundant group of proteins, also contributes to the yield of adducted Tyr and Cys residues, HSA and IgG were extracted from VX-exposed plasma separately by an affinity-based SPE workflow.

### Affinity-based SPE for IgG and HSA fractionation from plasma

The extraction kit used is typically applied to plasma samples prior to SDS-PAGE analysis to remove the most abundant proteins that deteriorate, e.g., electrophoretic separation and detection of low abundant proteins. HSA is extracted with the cibacron blue dye and IgG with protein A both immobilized on a resin packed into one cartridge, each. However, we used this extraction technique to isolate HSA as well as IgG for subsequent analysis of the extracted proteins similar to bioanalytical workflows described previously [32, 33].

**Fig. 6:**
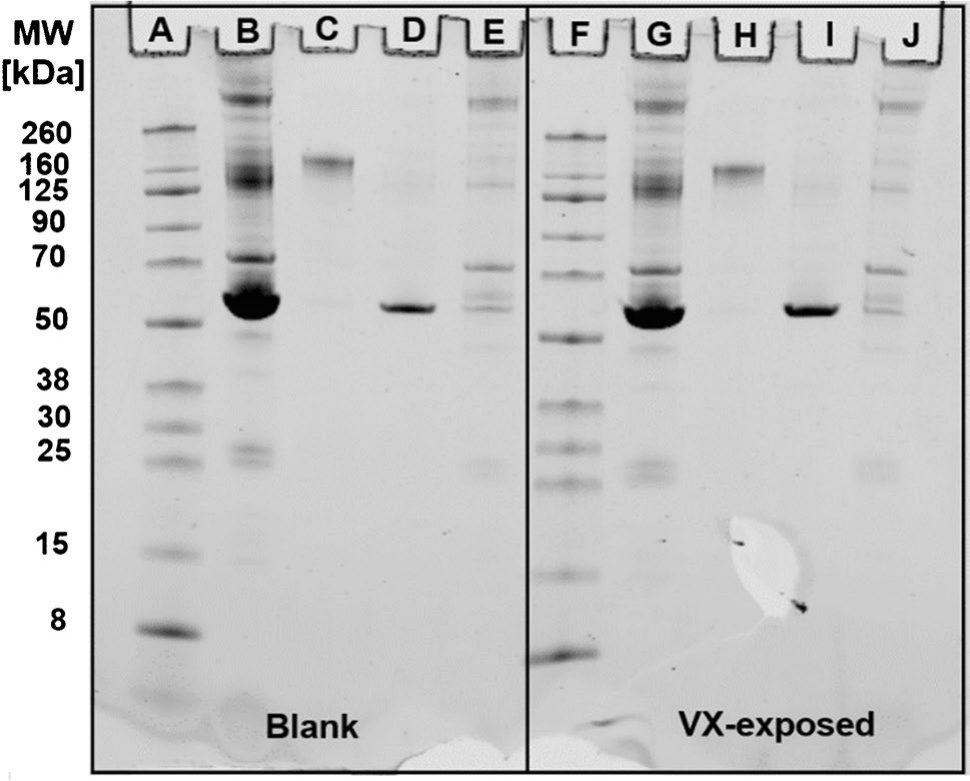
SDS-PAGE analysis of SPE fractions of plasma proteins. Lanes A to E: blank plasma. Lane A: molecular weight marker, lane B: total blank plasma, lane C: IgG fraction, lane D: HSA fraction, lane E: depleted fraction. Lanes F to J: VX-exposed plasma. Lane F: molecular weight marker, lane G: total plasma reference, lane H: IgG fraction, lane I: HSA fraction, and lane J: depleted fraction. Dominant bands at approximately 150 kDa (lanes C and H) and at 68 kDa (lanes D and I) represent IgG and HSA, respectively. Proteins were stained with Coumassie Brilliant Blue

The success of extraction and the purity of extracted HSA and IgG were documented by SDS-PAGE. Bands of highly purified proteins at approximately 150 kDa (Fig. 6 lanes C and H) and at 68 kDa (Fig. 6 lanes D and I) represent IgG and HSA, respectively. These bands were found in both blank plasma (Fig. 6, left side) as well as VX-exposed plasma (Fig. 6, right side), thus documenting the suitability of affinity-based SPE for protein adduct extraction.

The identity of the proteins as well as the presence of adducts in VX-exposed plasma was proven after in-gel enzymatic cleavage. After VX exposure, Tyr(-*EMP*) and Cys(-*DPAET*) were detected in both the HSA and the IgG band proteolyzed with pronase, thus proving that both biomarkers originated from both proteins.

As this affinity-based SPE extraction procedure might also be used for plasma sample preparation for the verification of exposure, the SPE step was characterized with respect to extraction efficacy, recovery, and reproducibility.

### Sample preparation by affinity-based SPE

HSA (40 mg/mL) and IgG (16 mg/mL) are present in plasma in very high concentrations. Therefore, sensitive µLC-ESI HR-MS/MS methods do not require the quantitative recovery of the proteins after affinity-based SPE. Accordingly, only the first 2 mL of the respective SPE elution volumes (4 mL in total, each) was collected and used for further preparation (HSA and IgG fraction). For the subsequent proteolysis, it was required to exchange the respective elution buffers by 50 mM NH_4_HCO_3_ known to be best suited for pronase treatment. This buffer exchange was realized by repeated UF (MWCO 10 kDa) adding NH_4_HCO_3_ buffer to the respective remaining retentates, each. Following this procedure, the proteins were washed to discard any small molecule as well as concentrated to obtain easy to manage volumes for proteolysis. The resulting concentrations obtained in triplicate were determined by a commercial BCA assay and found at 7.8 ± 1.1 mg/mL (RSD 14%) for HSA and at 3.5 ± 0.4 mg/mL (RSD 12%) for IgG (*n* = 3, each) correlating to recoveries of 48.6% ± 6.8% for HSA and 54.7% ± 6.4% for IgG. Even though these recoveries might appear to be quite small, the absolute amounts of extracted proteins (1.9 ± 0.3 mg for HSA and 0.88 ± 0.10 mg for IgG) as well as the reproducibility of extraction and concentration were highly suited for further analysis.

**Fig. 7:**
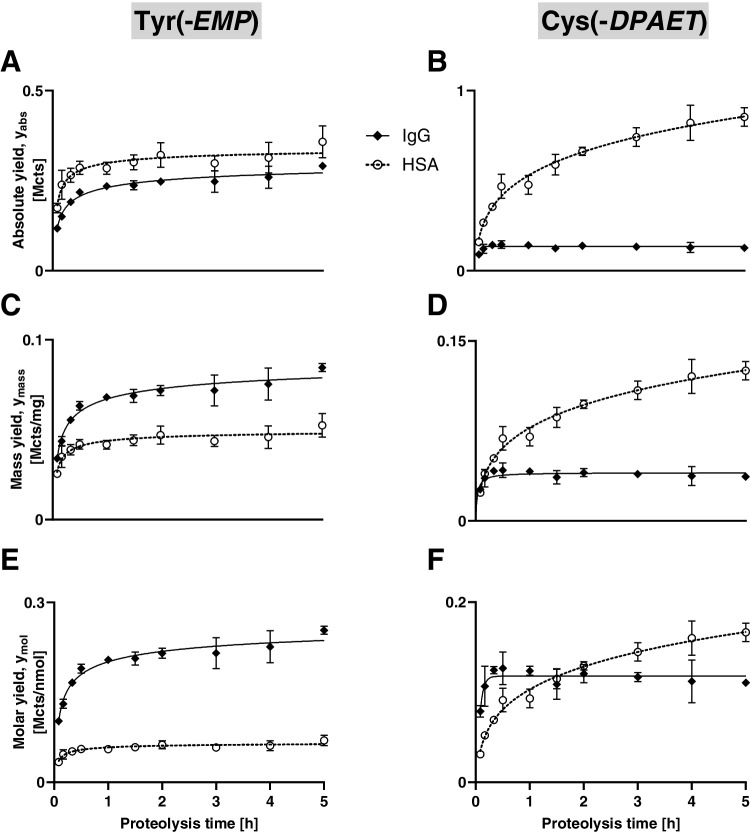
Time-dependent biomarker yield during proteolysis of proteins from SPE fractions. **A** Absolute yield (y_abs_) of Tyr(-*EMP*), **B** y_abs_ of Cys(-*DPAET*), **C** mass yield (*y*_mass_) of Tyr(-*EMP*), **D** *y*_mass_ of Cys(-*DPAET*), **E** molar yield (*y*_mol_) of Tyr(-*EMP*), and **F** *y*_mol_ of Cys(-*DPAET*). The biomarker yield was monitored recording Qual I of Tyr(-*EMP*) (*m/z* 214.063 ± 0.005 Th) and Cys(-*DPAET*) (*m/z* 151.983 ± 0.005 Th), with IgG fractions as filled diamonds with solid line and HSA fractions as open circles with dotted line. Data points represent *M* ± SD of triplicate analysis

After extraction of HSA and IgG from VX-exposed plasma, their fractions were subjected to proteolysis to monitor the time-dependent formation of both biomarkers (Fig. 7).

### Measurement of adduct yield during proteolysis of SPE fractions

Initially, the time-dependent absolute peak areas of Tyr(*-EMP*) and Cys(-*DPAET*) obtained from pronase-catalyzed protein cleavage were used as a measure of the biomarker yield. This yield is referred to herein as y_abs_ and illustrated in Fig. 7A for Tyr(*-EMP*) and in Fig. 7B for Cys(*-DPAET*). To compare the relative yields of Tyr(-*EMP*) and Cys(*-DPAET*) obtained from the HSA fraction with those obtained from the IgG fraction two additional protein-specific measures were introduced. To correlate the biomarker peak areas to the amount of the respective protein that was subjected to proteolysis, the “mass yield” was calculated (mass given in mg; y_mass_ = peak area/protein amount [Mcts/mg]). The corresponding time-dependent profile is depicted in Fig. 7C for Tyr(-*EMP*) and in Fig. 7D for Cys(*-DPAET*). In addition, the correlation of peak areas to the initial molar amount of proteins was described by the “molar yield” (molar amount given in nmol; y_mol_ = peak area/protein amount [Mcts/nmol]). For calculation of the molar amount the molecular weight of HSA was considered as 66,500 g/mol and that of IgG as 150,000 g/mol. This time-dependent profile of y_mol_ for Tyr(-*EMP*) is illustrated in Fig. 7E and that for Cys(*-DPAET*) in Fig. 7F. y_mass_ and y_mol_ allow the comparative characterization of the biomarker formation under consideration of the different protein concentrations and different molecular weights, thus shedding more light on potential structure-related differences.

The different time-dependent yields are discussed as follows:

### Yield of Tyr(-*EMP*)

Y_abs_ of Tyr(-*EMP*) from both the IgG fraction and the HSA fraction rose constantly within the first hour of proteolysis and then reached a plateau after 2 h (Fig. 7A). y_abs_ of Tyr(-*EMP*) obtained from HSA fractions was slightly higher than that obtained from the IgG fractions (Fig. 7A) indicating a slightly higher absolute concentration of this biomarker. In contrast, y_mass_ of Tyr(*-EMP*) from the IgG fraction (Fig. 7C) was about 30% higher than that of the HSA fraction. This ratio clearly documents IgG as a valuable source of Tyr(*-EMP*) being more productive than HSA. This fact is even more obvious when calculating y_mol_ as illustrated in Fig. 7E. y_mol_ of the IgG fraction was about three times higher than that of the HSA fraction. The primary structure of HSA contains 18 Tyr residues in total, from which merely about seven were shown to be prone to phosphylation [32]. In contrast, the constant regions of the most abundant chains of IgG alone (heavy chain γ1 and light chain κ UniProt IDs P01857 and P01834, respectively) contain 32 Tyr residues in total. Additional Tyr residues being part of the variable region of the IgG molecule might also contribute to the yield of Tyr(*-EMP*). Therefore, it was shown that (i) IgG is a highly relevant source of Tyr(*-EMP*) when analyzing VX-exposed plasma and (ii) a certain number of Tyr residues in IgG exhibits a sufficient reactivity.

### Yield of Cys(-*DPAET*)

Y_abs_ of Cys(*-DPAET*) obtained from the IgG fraction reached its plateau after about 45 min of proteolysis (Fig. 7B). In contrast, y_abs_ obtained from the HSA fraction showed a steep increase within the first 2 h and continued rising afterwards reasonably (Fig. 7B). The HSA-related profile documents the onholding release of Cys(*-DPAET*). This phenomenon might be the result from the progressing degradation of adducted Cys-containing peptides that are initially produced by pronase-catalyzed cleavage. Even though it was shown that the adducted dipeptide Cys^34^Pro from HSA was stable for at least 7 h of proteolysis [20, 33, 34], the final cleavage of Cys^34^ and release of other Cys residues appear quite likely. Furthermore, it is expected that Cys(*-DPAET*) might result from intramolecular disulfide exchange causing the transfer of the *DPAET*-group from any adducted Cys-containing peptide to the single Cys residue. Comparable effects were observed for diverse mercaptan adducts produced in human plasma after in vitro incubation [35, 36]. However, y_abs_ of Cys(*-DPAET*) obtained from HSA was about seven times higher than that obtained from IgG (Fig. 7B), thus indicating that HSA will play the major role for verification.

A very similar relation was obvious from the time course of y_mass_ underlining the major impact of HSA (Fig. 7D). A quite different picture results from the time profile of y_mol_ (Fig. 7F). Within the first 1.5 h of proteolysis, y_mol_ of Cys(*-DPAET*) obtained from IgG was much higher than that obtained from HSA (Fig. 7F) indicating a rapid and maximum release of adducted residues. However, after 2 h, y_mol_ from HSA further increased and exceeded that of IgG most presumably due to the higher number of Cys residues found in HSA. After 5 h, y_mol_ for IgG was only about 75% of that from HSA. IgG contains a total of 12 intramolecular disulfide bridges in the constant regions of heavy chain γ1 and the light chain κ as well as an undefined number of non-disulfide-bridged Cys residues (24 Cys residues at least in total). In contrast, HSA contains 17 intramolecular disulfide bridges as well as one highly reactive non-bridged residue (Cys^34^) (35 Cys residues in total). Although being bridged, some Cys residues might be prone to disulfide exchange as shown previously: Bridged Cys residues might undergo *S*-thiolation reactions with, e.g., cysteine and homocysteine [37] as well as the V-type nerve agent leaving groups [19, 20] and malodorous mercaptans [35]. However, in summary, both the IgG and HSA fractions were shown to be well suited for the generation of both biomarkers.

## Conclusions

In addition to the well-characterized HSA, we herein elaborated that IgG as the second most abundant protein in plasma also contributes reasonably to the total yield of phosphonylated Tyr residues and disulfide-adducted Cys residues. The single Cys residue adduct with the VX leaving group is shown to be well suited as a biomarker of exposure to VX at least in vitro.

The affinity-based SPE workflow did not only help to confirm IgG as a source of biomarkers but may also represent a valuable tool for biomedical verification. As Tyr(*-EMP*) and Cys(*-DPAET*) originate from different separated plasma proteins, they both might be considered as primary biomarkers from HSA as well as IgG as defined by the OPCW guidelines [23]. Future studies will focus on additional adducts of, e.g., single amino acids as already described for Lys(*-EMP*) from hair keratins [16] or of small dipeptides as may be produced from HSA or BChE. The same strategy might also be transferred to other V-type nerve agents like Russian VX and Chinese VX as well as to plasma derived from non-human species whose serum albumin as well as IgG might be separated by the same or slightly modified SPE columns.

In summary, the presented results shed more light on the molecular toxicology of VX, provide a valuable tool for plasma sample preparation, and broaden the spectrum of biomarkers targeted for biomedical verification.

## Abbreviations

AChE: Acetylcholinesterase
ACN: Acetonitrile
BCA: Bicinchoninic acid
BChE: Butyrylcholinesterase
CE: Collision energy
CID: Collision-induced dissociation
CWA: Chemical warfare agent
CWC: Chemical Weapons Convention
Cys(-*DPAET*): Disulfide adduct of cysteine and the *DPAET* leaving group of VX
Cys: Cysteine
*d*_3_-Atr: Triple deuterated atropine
*DPAET*: 2-Diisopropylaminoethanethiol moiety
EDTA: Ethylenediaminetetraacetic acid
*EMP*: Ethyl methylphosphonyl moiety
ESI: Electrospray ionization
FA: Formic acid
HR-MS/MS: High-resolution tandem-mass spectrometry
HSA: Human serum albumin
IgG: Immunoglobulin G
μLC: Microbore liquid chromatography
LOI: Limit of identification
Lys: Lysine
*M*: Mean
[M + H]^+^: Single protonated precursor ion
MS: Mass spectrometry
MWCO: Molecular weight cutoff
OPCW: Organisation for the Prohibition of Chemical Weapons
OPNA: Organophosphorus nerve agent
PBS: Phosphate-buffered saline
PIS: Product ion scan
Pro: Proline
Qual: Qualifying ion
RCF: Relative centrifugal force
*RSD*: Relative standard deviation
*SD*: Standard deviation
SDS-PAGE: Sodium dodecyl sulfate–polyacrylamide gel electrophoresis
SPE: Solid-phase extraction
TOF: Time-of-flight
t_R_: Retention time
Tyr(-*EMP*): Tyrosine phosphonylated by *EMP* of VX
Tyr: Tyrosine
UF: Ultrafiltration
XIC: Extracted ion chromatogram
y_*abs*_: Absolute yield (given as peak area)
*y*_*mass*_: Mass yield (given in peak area/mg protein)
*y*_*mol*_: Molar yield (given as peak area/nmol protein)

## Declarations

### Source of biological material

Plasma was obtained from commercial sources. Research was carried out without human participants and animals.
